# Correlation of X-Ray Computed Tomography with Quantitative Nuclear Magnetic Resonance Methods for Pre-Clinical Measurement of Adipose and Lean Tissues in Living Mice

**DOI:** 10.3390/s141018526

**Published:** 2014-10-08

**Authors:** Matthew N. Metzinger, Bernadette Miramontes, Peng Zhou, Yueying Liu, Sarah Chapman, Lucy Sun, Todd A. Sasser, Giles E. Duffield, M. Sharon Stack, W. Matthew Leevy

**Affiliations:** 1 Department of Chemistry and Biochemistry, 236 Nieuwland Science Hall, University of Notre Dame, Notre Dame, IN 46556, USA; E-Mails: mmetzin1@nd.edu (M.N.M.); yliu12@nd.edu (Y.L.); sstack@nd.edu (M.S.S.); 2 Harper Cancer Research Institute, A200 Harper Hall, University of Notre Dame, South Bend, IN 46617, USA; E-Mail: luciesun101@gmail.com; 3 Department of Biological Sciences, Galvin Life Sciences Center, University of Notre Dame, Notre Dame, IN 46556, USA; E-Mails: bmiramon@nd.edu (B.M.); pzhou@nd.edu (P.Z.); gduffiel@nd.edu (G.E.D.); 4 Notre Dame Integrated Imaging Facility, 416c Main Building, University of Notre Dame, Notre Dame, IN 46556, USA; E-Mails: sarah.chapman@nd.edu (S.C.); todd.sasser@carestream.com (T.A.S.); 5 Department of Nutritional Science and Toxicology, 119 Morgan Hall #3104, University of California, Berkeley, CA 94720, USA

**Keywords:** X-ray CT, microCT, computed tomography, QMR, quantitative magnetic resonance, adipose tissue, body composition, obesity

## Abstract

Numerous obesity studies have coupled murine models with non-invasive methods to quantify body composition in longitudinal experiments, including X-ray computed tomography (CT) or quantitative nuclear magnetic resonance (QMR). Both microCT and QMR have been separately validated with invasive techniques of adipose tissue quantification, like post-mortem fat extraction and measurement. Here we report a head-to-head study of both protocols using oil phantoms and mouse populations to determine the parameters that best align CT data with that from QMR. First, an *in vitro* analysis of oil/water mixtures was used to calibrate and assess the overall accuracy of microCT *vs.* QMR data. Next, experiments were conducted with two cohorts of living mice (either homogenous or heterogeneous by sex, age and genetic backgrounds) to assess the microCT imaging technique for adipose tissue segmentation and quantification relative to QMR. Adipose mass values were obtained from microCT data with three different resolutions, after which the data were analyzed with different filter and segmentation settings. Strong linearity was noted between the adipose mass values obtained with microCT and QMR, with optimal parameters and scan conditions reported herein. Lean tissue (muscle, internal organs) was also segmented and quantified using the microCT method relative to the analogous QMR values. Overall, the rigorous calibration and validation of the microCT method for murine body composition, relative to QMR, ensures its validity for segmentation, quantification and visualization of both adipose and lean tissues.

## 1. Introduction

Obesity is defined as a body mass index (BMI) of ≥30, while a BMI of 25–29.9 is categorized as overweight [1]. In the United States of America, greater than one-third of the adult population is clinically obese [2]. Further, it is projected that 42% of the total US population will be classified as clinically obese in 2030, a 33% increase from 2010 [3]. However, the World Health Organization (WHO) reports that obesity and overweight populations are not only problems for high-income countries, but also those with low- and middle-incomes [1]. Additionally, incidence rates for several clinical conditions, such as type 2 diabetes, heart disease, stroke, and some types of cancer increase due to obesity [4,5], suggesting a clinically relevant co-morbidity. Research efforts into obesity and its clinical consequences have dramatically increased in recent years, and corresponding mouse models have been developed for these purposes. For example, the B6.V-Lepb and lethal yellow mutant mouse (Ay) lines are routinely used for obesity studies in small animals [6]. In addition to genetic models, diet-induced obesity (DIO) rodent models have also been developed, and are based predominantly on consumption of a high fat and/or high caloric content diet [7].

Pre-clinical research using murine obesity models has been vastly enhanced through the use of non-invasive anatomical imaging methods. X-ray computed tomography (CT) and quantitative nuclear magnetic resonance (QMR) are two techniques that enable the measurement of adipose tissue in these mouse models. QMR is a type of magnetic resonance imaging (MRI) that measures the masses of fat tissue, lean tissue, free water, and total water [8]. QMR has been used on both human [9] and animal subjects [10], and functions by measuring the entire body at once [8]. X-ray CT is a highly utilized imaging technique for the non-invasive and quantitative analysis of dense structure like bone, and may be readily applied to also study body composition. In the pre-clinical setting, microCT instruments have been developed with excellent resolution to visualize small animal anatomy with manageable specimen radiation doses [11]. Briefly, the technology relies on the acquisition of a series of planar X-ray images captured at various angles around a given specimen using a gantry based system. This data is input into a filtered back projection algorithm to yield a three dimensional array of radiodensity values and computational segmentation tools may be used to selectively extract the adipose volume using its innate contrast properties [12].

The clinical and pre-clinical validation of CT and microCT are critical to their utilization in each respective setting. In the clinical setting, patient adipose values obtained using MRI-based T_1_-mapping have been correlated with CT and dual energy X-ray absorptiometry (DEXA) imaging modalities [13]. Meanwhile, microCT has been validated for pre-clinical use in adipose tissue quantification by *ex vivo* adipose tissue extraction and measurement [14,15]. However, a direct comparison of QMR and microCT in small animals has, to our knowledge, not been reported. This is of particular importance given the increased sensitivity and resolution needed to detect and measure fat deposits within pre-clinical specimens. In the current study, we quantitatively compare a microCT imaging and segmentation method for adiposity quantification with data obtained using QMR. Data were collected using both platforms in an *in vitro* study with oil and water mixtures, and an *in vivo* study using two different strains of mice. Further, we investigate the specific parameters that best align microCT data with that from QMR.

## 2. Materials and Methods

### 2.1. Oil and Water Sample Preparation

A series of 50 mL Falcon tubes were prepared containing 25 g of water with varying amounts of soybean oil (0, 3, 6, 9, 12, 15, and 18 g) using the Denver Instrument MXX-612 (Denver Instrument, Bohemia, NY, USA) analytical balance. Each series was prepared in triplicate, such that three samples of each oil/water mixtures were scanned and analyzed.

### 2.2. Mouse Models

The experiment was conducted using two different cohorts of mice. Cohort A was composed of 28 nude female mice (NU/NU, Foxn1 knockout, age = 16 weeks) and Cohort B was composed of 17 total male (*n* = 9) and female (*n* = 8) *Id2*−/− and *Id2*+/+ mice on a mixed strain background (129sv/C57BL6J/FBVN/CD1) [16,17] and aged 13–37 weeks. Inhibitor of DNA binding 2 (ID2) is a rhythmically expressed transcriptional repressor required for circadian clock output in the mouse liver [16,18]. As *Id2* null mice exhibit a discrete adipogenic phenotype, we used these mice as a model as they exhibit low adiposity compared to their WT littermates [17,18], thereby offering a broader range of adipose mass to evaluate within a single population.

### 2.3. X-Ray MicroCT Scans

The *in vitro* microCT experiments were conducted on the Albira CT System (Bruker BioSpin Corporation, Billerica, MA, USA) based on published protocols [19]. Two scans of each oil and water mixture were acquired at 45 kVp, with currents of 0.4 (high dose) and 0.2 (low dose) mA, at a 155 mm field of view (FOV). Acquisitions of 600 projections were taken for each tube and a 125 μm isotropic voxel size (high resolution) image was reconstructed via filtered back projection. Note that isotropic voxels are used for all data sets reported herein, with the “size” reported as one edge of the voxel cube. For studies with animals, the mice were anesthetized with isofluorane (2.5% flow rate) both prior to and during the scans. Scans of the mice were performed with a FOV of 115 mm at low dose CT intensity (0.2 mA) and a high CT voltage (45 kVp). The initial horizontal position of the mice on a standard rat bed was 15.00 mm and 400 projections were taken for each mouse as defined by the “Good” CT setting. The decision to scan the mice using the “Good” CT setting was made to minimize radiation exposure to the mice during this longitudinal study. The raw projection data was reconstructed three separate times using voxel sizes of 500, 250, and 125 μm under standard conditions with the Albira Suite 5.0 Reconstructor (Bruker BioSpin Corporation, Billerica, MA, USA) with the data output in Hounsfield units (HU). Additional reconstructions of 10 mouse scans were completed at both low (500 μm voxel size) and high (125 μm voxel size) resolutions for analysis of adipose and lean tissue.

### 2.4. X-Ray MicroCT Analysis

Analysis of the microCT scans of the oil and water samples was conducted using the PMOD software (PMOD Technologies LTD, Zurich, Switzerland). Each image corresponding to the set with varying amounts of soybean oil was segmented using the range of −300 to −50 HU to select for the soybean oil in the mixture and the plastic tube in which the mixture was contained, thereby leaving out the water. Each image was segmented with a range of −50 to +110 HU to select for the water in the mixture, thereby leaving out the soybean oil and plastic tube. These segmented regions were then quantified to produce a volume in cm^3^. Empty plastic tubes were also scanned under the same conditions and segmented within the −300 to −50 and −50 to +110 ranges to determine any background values associated with plastic, which is constructed of hydrocarbons that will have similar density to fatty tissue. The background volumes of the plastic itself (−300 to −50 HU) were subtracted from the volumes of the soybean oil with plastic in order to quantify only the volume of the soybean oil in each sample. After completing the same steps for each of the three samples for each oil amount, the average volume of soybean oil was calculated. Multiplying each average volume by the density of soybean oil (0.926 g/mL) gave the calculated masses of soybean oil in each sample based on the microCT segmentation and quantification.

Analysis of the microCT scans of the mice was performed mostly as adapted from Sasser *et al.* [19]. The method is described briefly here. The reconstructed files were opened in the PMOD software (PMOD Technologies LTD, Zurich, Switzerland) and then segmented based on the varying densities of adipose and tissue. The number of frames in each of the *x*-, *y*-, and *z*-planes was reduced by a factor of two. Next, selection using the volume of interest (VOI) tool was completed on each of the remaining slices so that only the body of the mouse was selected for extraction. All items outside of this selected VOI such as the bed were masked out using a value of −1000. Segmentation with a range of −300 to +3500 HU was then performed on this VOI in order to calculate the total body volume of the mouse in cm^3^. The VOI containing the entire mouse body was then segmented with a range of −300 to −50 HU to extract only the fat tissue within the mouse. Note that the Erosion and Dilation options were not subsequently used. The mass of body fat in each mouse was calculated by multiplying the volume of body fat by 0.90 g/mL, the density of adipose tissue [20]. Lean tissue (muscle, internal organs) volumes were segmented using the range of −50 to +285 HU, which was chosen in order to avoid collecting adipose tissue and bone. The lean tissue volumes were then multiplied by 1.05 g/mL to find the mass of lean tissue.

### 2.5. QMR Scans

QMR scans of the soybean oil and water samples were performed using the EchoMRI-500 QMR system (EchoMRI, Houston, TX, USA). A system test with a tube of 38.4 grams of canola oil was run in order to calibrate the system for mice. Each tube of the soybean oil and water mixture was placed in a plastic tube sized for rats that was then inserted into the QMR machine for scanning. Each tube was scanned three times with a 40 s pause between scans, and values for the masses of fat tissue, lean tissue, free water, and total water were recorded. For mouse scanning, the mass of each specimen was recorded in grams using a Denver Instrument MXX-612 (Denver Instrument Bohemia, NY, USA) analytical balance before scanning. Each mouse was then placed individually into a plastic tube (30 and 40 gram sizes) and its movement was prevented by inserting a smaller plastic tube to hold the mouse at the end of the tube for scanning. Each mouse was scanned three times with a 40 s pause, a primary accumulation of 1 and a water stage. Mass measurements for fat tissue, lean tissue, free water, and total water were then produced for all scans in the EchoMRI Body Composition Analyzer EMR-184 software (EchoMRI, Houston, TX, USA).

### 2.6. QMR Analysis

Averages for each set of fat mass values, with standard error of the mean (SEM) for each tube, were calculated and plotted against the fat mass values obtained from the microCT scans. The same was done for the fat mass values calculated from the QMR mice scans. Lean tissue mass values were calculated in the same manner. Graphical figures were produced and linear regression was performed using Graphpad Prizm v5.

## 3. Results

### 3.1. In Vitro Validation Study of MicroCT and QMR

**Figure 1.**
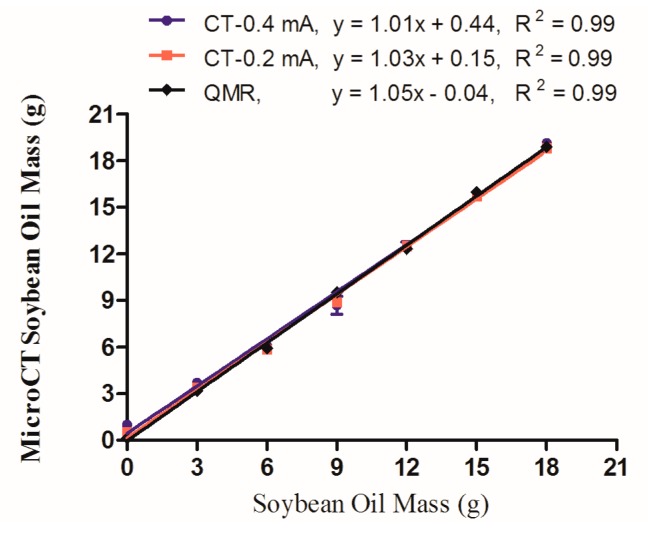
*In vitro* measurement of soybean oil masses as measured by QMR and microCT (0.4 and 0.2 mA currents) methods.

An initial *in vitro* study was conducted to verify the accuracy of the QMR and microCT techniques in a rigorously controlled experimental setup. Oil/water dilution samples were prepared in triplicate, in which 0, 3, 6, 9, 12, 15, and 18 g of soybean oil were mixed with 25 g of water in a 50 mL Falcon tube. This range of oil mass was chosen to mimic the typical adipose content reported for living mice within a similar total volume. Each sample was scanned using both instruments, but in the case of microCT the samples were scanned with both the “high” (0.4 mA) and “low” (0.2 mA) current settings to test its effect on the output values. Using the segmentation range of −300 to −50 HU for microCT data, and the QMR mass calculation of fat tissue, the averages (with standard error of the mean, SEM) for each dilution were plotted in Figure 1. In each case, the measured values gave an excellent linear fit with a slope of 1 and R^2^ values of 0.99. The microCT-0.4 mA, microCT-0.2 mA and QMR had y-intercept values of 0.44, 0.15, and −0.4, respectively.

### 3.2. In Vivo Adipose Measurements with MicroCT and QMR

A longitudinal *in vivo* study was executed to determine the correlation between QMR and microCT in living mice. A cohort of 28 athymic nude mice (female, strain Nu/Nu) was scanned using both the microCT and QMR methods at three separate time points over the course of 18 weeks. With microCT, we utilized the Albira platform with 45 kVp voltage, 0.2 mA current and a 0.5 mm Al filter over the source, which is in alignment with previous reports of using CT to measure adipose in small animals [21]. The first joint scanning was performed on the first day of the study in which the mice were 16 weeks of age, with the second and third time points conducted after 6 weeks (22 weeks of age) and 18 weeks (34 weeks of age), respectively. Of these 28 mice, the 16 with the highest relative weight gain were selected for extensive analysis of their microCT data, yielding 48 values with the widest range available for plotting. The microCT data was examined to determine the effects of varied adipose segmentation ranges, data filters, and reconstruction resolution on both linearity and correlation with QMR data; results are detailed below.

#### 3.2.1. MicroCT Image Resolution

**Figure 2.**
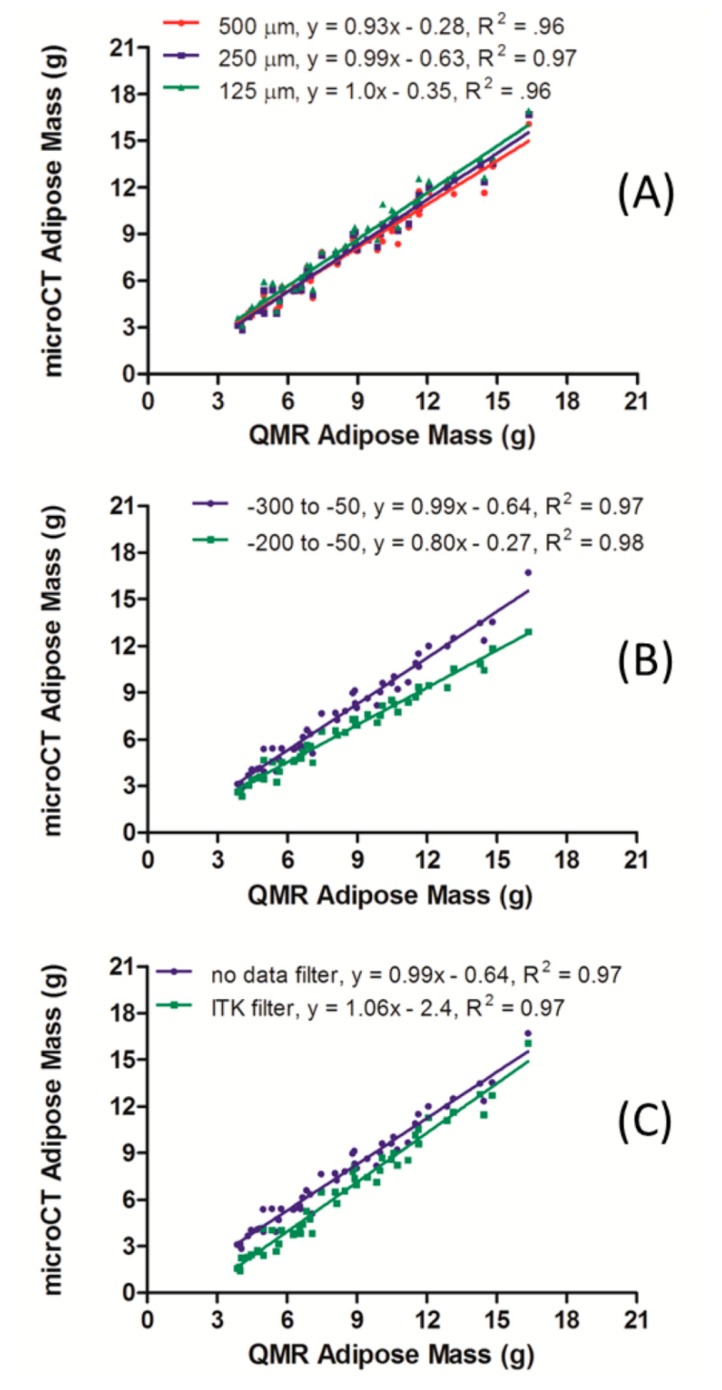
Effects of resolution (**Panel A**), segmentation range (**Panel B**) and median filter (**Panel C**) on microCT data alignment with QMR.

MicroCT scanners will acquire and reconstruct a whole mouse at a variety of resolutions depending on the combination of system hardware and setting variables. In general, whole mouse data sets have a minimum voxel size (maximum resolution) in the range of 100 μm. This value provides an optimum balance of radiation dose, data size, and field-of-view, and can meet the majority of application demands to image fat, as well as lung, liver, kidney, GI, and other soft tissues [12]. However, some scanners, due mostly to technical hardware limitations, may not provide images suitable for all applications. To test the effect of resolution on fat segmentation and quantification, the 48 microCT scans from 16 mice (detailed in Section 3.2) were reconstructed at three resolutions with 500, 250 and 125 μm isotropic voxels. Each of these scans was segmented for total body volume adipose tissue (−300 to −50 HU) and multiplied by a density of 0.90 g/mL to yield a mass. These results were then plotted against the mass outputs from the QMR scans (Figure 2A). In each case, the linearity fit yielded an R^2^ value over 0.96. While the 125 and 250 μm data sets did have a slope equal to 1, the 500 μm yielded a slightly lower value of 0.93.

#### 3.2.2. MicroCT Segmentation Range

Adipose segmentation parameters were evaluated to determine the best radiodensity range that best aligns microCT and QMR data. Quantification of adipose tissue from the medium resolution (250 μm isotropic voxel size) microCT scans was performed based on the segmentation ranges of −200 to −50 HU and −300 to −50 HU. The aggregate data from these studies were compiled, and each adipose mass value derived from microCT was paired with the corresponding measurement from QMR. A plot of both segmentation ranges for the nude mouse study is shown in Figure 2B. The linearity between the microCT and QMR values derived from using both segmentation methods is illustrated in the two R^2^ values, both of which were greater than 0.97. The precision of the measurements between the two methods, however, was different depending on the segmentation range used. The slope of 0.99 in the −300 to −50 HU range illustrates the stronger linear fit of the two segmentation ranges but still underestimates the adipose mass value by an average of 0.64 g at each weight relative to QMR measurements.

#### 3.2.3. MicroCT Median Filter

In some cases, a user may wish to smooth a segmented adipose data set using a median filter. In short, this filter will take a given voxel and examine a user-specified number of adjacent voxels in the *X*, *Y* and *Z* directions. The voxel value will then be set to the median of the range. This type of data filtration is used to eliminate potential image artifacts like lines or other small irregularities. An assessment of the effect of median filtration was performed on data reconstructed at a 250 μm voxel size. Note that during image processing, each CT data set was reduced by a factor of 2 on each axis since PMOD was unable to otherwise computationally handle the data sets. Thus, after image processing and segmentation with a range of −300 to −50 HU, an adipose data set was produced in which each adipose positive voxel had a value of 1, and a 500 μm size. The median filter, within the ITK menu on PMOD, was then applied using a distance of 1 mm in the *X*, *Y*, and *Z* directions as shown in Figure 2C. The linear fit for the median filter data was strong with an R^2^ of 0.97 and a slope of 1.06. However, this line crossed the *y*-axis at −2.4, meaning that the median filter caused the fat content to be underestimated relative to the QMR. The linear fit for the data not subjected to median filter was equally strong with an R^2^ of 0.97 and a slope of 0.99. As with the median filter data set, this set's y-intercept of −0.64 indicates that there is an underestimation of the microCT adipose mass compared to that of the QMR method.

### 3.3. MicroCT Imaging of Lean Tissue

**Figure 3.**
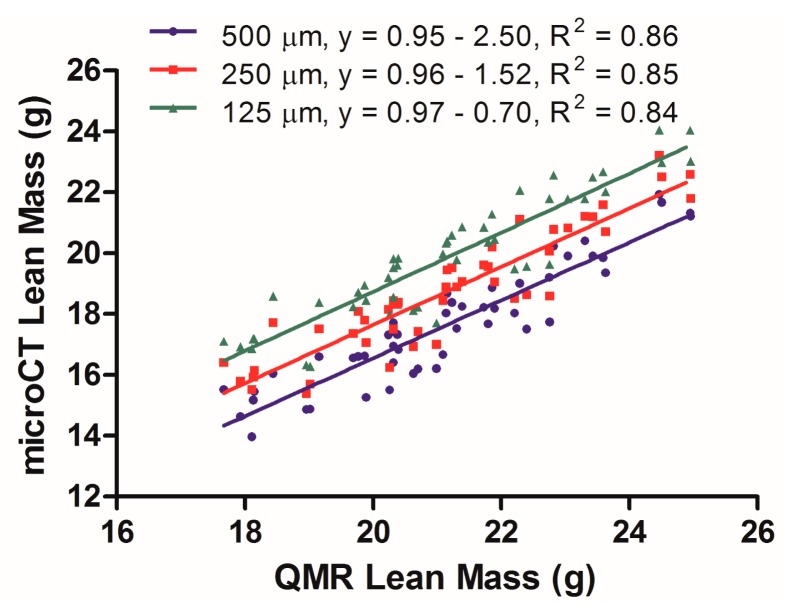
Linear regression of lean tissue values derived from QMR *vs.* microCT data at 500, 350 and 125 μm voxel size.

A separate analysis was conducted to assess the correlation between microCT and QMR for lean tissue quantification. Further, the effect of resolution on microCT calculation of lean tissue (muscle, internal organs) was evaluated. Three mouse data sets reconstructed at 500, 250 and 125 μm, each containing 16 mice scanned at the 3 time points, were segmented within the range of −50 to +285 HU in order to select for lean tissue. HU values larger than +285 HU started to include some peripheral bone structure in the resulting images and were thus excluded. The resultant lean tissue volumes were then multiplied by the density of 1.05 g/mL and plotted in Figure 3 with the corresponding mass values gathered from QMR scans. In terms of lean tissue segmentation and quantification, the microCT method has a reduced correlation when compared to the QMR technique, as evidenced by linear regression analysis (Figure 3) that yielded an R^2^ value between 0.84 and 0.86. Further, microCT lean tissue quantification was sensitive to reconstruction quality, as each increase in voxel size decreased the *y*-axis intersection values from −0.70, to −1.52, to −2.50, resulting in a wider systematic difference with the QMR results. The microCT method also consistently yielded lean mass values that were lower than those measured by QMR, as evidenced by the negative y-intercept values in the linear regression.

### 3.4. QMR and MicroCT Adipose Measurements of a Heterogeneous Mouse Population

**Figure 4.**
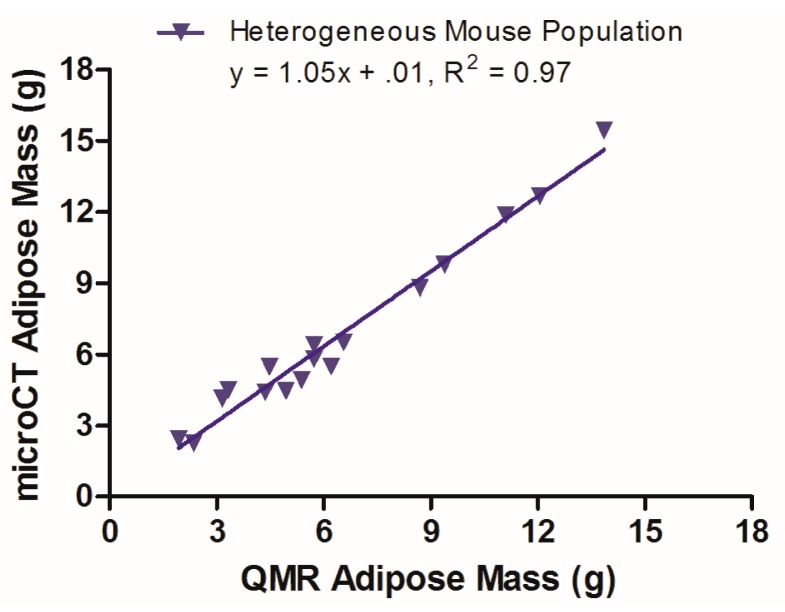
Linear regression of microCT and QMR adipose values for a mouse population with heterogenous genetic background.

A mixed population of mice (male and female *Id2*−/− or *Id2* WT mice with a mixed genetic background [129sv/C57BL6J/FBVN/CD1] and aged 3–9 months) was used to test the optimum microCT imaging conditions developed in studies of the homogenous nude mouse population. A total of 17 mice were scanned with microCT with an output voxel size of 250 μm. Analysis was performed using a segmentation range of –300 to –50 HU, and no data filtering. Figure 4 presents the linear regression of the data from this study, with a slope of 1.05 and y-intercept of 0.01.

### 3.5. Total Body Mass

Analysis of the calculated masses for adipose and lean tissues, and the observed values measured via a balance, was completed in order to quantify the degree of difference seen in the measurements produced by each method. The adipose, lean and free water masses from the QMR were summed in order to find the total mass for the mice. A percent difference was calculated for all 48 data points, with QMR yielding a −3% (+/− 0.002, SEM) difference. The total body mass values derived from microCT were dependent on the reconstructed voxel size, with 500, 250, and 125 μm data sets yielding −18% (+/− 0.04, SEM), −14% (+/− 0.03, SEM), and −9% (+/− 0.03) respectively. When the skeleton was independently segmented (285–2000 HU) and added to the body mass total, the differences were reduced to −16%, −11%, and −6% respectively. Note that microCT images omitted the tails of the animals, which had an average weight of 0.5 g, or approximately 2% of body weight. Both the microCT and QMR methods underestimate the total body mass, but the difference between the QMR-derived and observed mass values were smaller than that noted with microCT.

### 3.6. Visualization of Whole Body Adipose and Lean Tissue Distribution from MicroCT Scans

**Figure 5.**
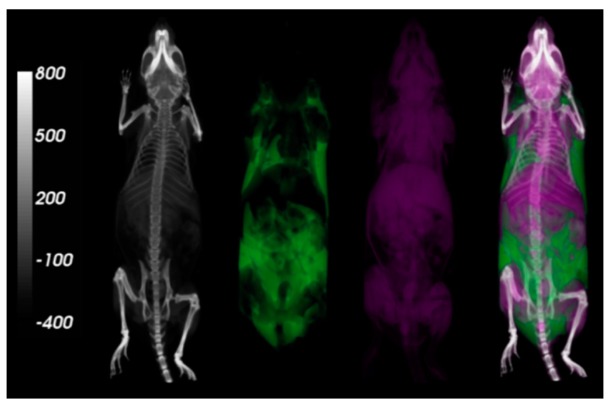
Three-dimensional visualizations of: (**a**) all skeletal and soft tissue; (**b**) region-specific adiposity; (**c**) lean tissue; and (**d**) an overlay of the three in a mouse from Cohort A at the 6 week time point.

Using the data from the microCT scans and analysis, 3D visualizations of whole body adipose distribution were created using the VolView software (Kitware, Clifton Park, NY, USA). These images, however, were reconstructed using “High Resolution” settings in order to achieve clarity in the images. The microCT scans of the segmented adipose and lean tissues were merged with the entire mouse body to produce the images shown in Figure 5. The calibration of the microCT method based on the QMR technique for adipose tissue segmentation and quantification also validates the 3D visualization of regional-specific adiposity, a major advantage held by the microCT method over the QMR technique.

## 4. Discussion

The key difference between measurements obtained by QMR and MRI is that QMR will directly output numerical body composition values for fat, lean, and water content, while MRI will output an anatomical image that may be subsequently analyzed for these parameters. QMR readings, performed by systems such as the EchoMRI-500 (EchoMRI, Houston, TX, USA), are based on the NMR characteristics of hydrogen bonds and the density of hydrogen nuclei in body tissues. Contrast between different types of tissues such as fat, muscle, and water is created based on these readings, and this information is used in turn to quantify the mass of each [22]. When compared to MRI methods for body composition analysis, QMR holds many advantages. Magnetic resonance imaging utilizes expensive and complex equipment that typically requires separate rooms and highly trained operators. Further, long acquisition times and complete immobilization of the animal subjects under anesthesia are necessary [8]. Alternatively, with QMR, a non-expert user can collect body composition data on live specimens in under five minutes and without the use of anesthesia. Additionally, the whole body data rapidly obtained using QMR agrees well with more labor intensive methods. In previously reported validation studies, the QMR method of fat quantification had a positive correlation with post-mortem whole-body carcass composition analysis (CCA) [10,23] and (DEXA) [8,24] in animal models. Positive correlations have also been reported when using QMR to quantify lean tissue mass in many animal models [8–10,22–26]. The QMR approach was considered more precise than both the CCA and DEXA methods [8], especially when measuring small quantities of fat [22] or small deviations caused by changes in diets [9]. QMR has been used for body composition analysis in several *in vivo* studies including birds [25], mice [10,22,24], piglets [8], rats [10], bats [26], and in longitudinal studies in which dietary adjustments are used to cause changes in body fat [27]. QMR is widely considered the gold standard for measurement of longitudinal changes in body fat and lean tissue, particularly in small animals. However, one shortcoming of QMR analysis is the lack of an accompanying 3D visualization that displays both adipose and lean tissue throughout the body.

X-ray CT is a well-known imaging technique for the non-invasive and quantitative analysis of dense structure like bone, and may be readily applied to also study body composition. In the pre-clinical setting, microCT instruments have been developed with excellent resolution to visualize small animal anatomy with manageable specimen radiation doses [11]. MicroCT equipment is widely available at many research universities due to its low cost and general ease of use. Briefly, the technology relies on the acquisition of a series of planar X-ray images captured at various angles around a given specimen using a gantry based system. This rotation data is input into a filtered back projection algorithm to yield a three dimensional array of radiodensity values in Hounsfield units (HU). Structures like bone, which are dense and electron-rich, will readily absorb X-rays to yield high contrast in the resulting tomographic images (+1000 to +3000 HU). Meanwhile, less dense regions like water or air will have far less attenuation with lower associated values of 0 or −1000 HU, respectively. Fortuitously for obesity researchers, adipose tissue maintains a unique HU range of approximately −300 to −50 HU, which may be leveraged for quantitative measurement. Computational segmentation tools may be used to selectively extract the adipose volume into a separate data set using its innate contrast properties [12]. Studies have also been conducted in which lean tissue volumes were segmented and quantified using a different HU range from that used for adipose tissue [14,28]. As a non-invasive and non-terminal technique, microCT imaging is both accurate and precise [14] when measuring adiposity and effective in longitudinal studies [15], much like the QMR method. However, microCT does require the use of anesthesia, and while scan times are 2–4 times shorter than MRI, they are 2–4 times longer than those in the QMR method. Nevertheless, this bandwidth may be re-gained through the use of bed systems that simultaneously scan 2–4 animals. MicroCT differentiates itself in that it merges quantitative analysis with a 3D visualization of region-specific adiposity within the specimen [19], an output that is not provided by the QMR technique, and is cumbersome to achieve with classic MRI.

### 4.1. In Vitro Studies

An initial assessment of the fat measurement capabilities of QMR and microCT was performed on an *in vitro* phantom system comprised of water and oil, demonstrating excellent linear correlation between the two methods. For these data sets, linear regression provided the critical outputs to evaluate the accuracy and robustness of each method of fat measurement. The R^2^ value provides a straightforward metric for the goodness of fit of the linear model, which is critical for monitoring changes that occur during longitudinal studies of animals. The slope provides the ratio at which the measured CT values correspond to the QMR fat mass, and the y-intercept reports any offset. Each approach yielded linearity with a slope of 1 and an R^2^ value of 0.99. However, the 0.4 mA high current setting did induce a slightly elevated offset of 0.44 at the y-axis, while the corresponding 0.2 mA setting was only 0.15, indicating that the higher current will overestimate fat content by up to 0.44 grams.

This initial study highlights the quantitative and semi-quantitative capabilities of the microCT method for fat analysis. When linearity is present in the output regression, the resulting values may be used in a semi-quantitative fashion to monitor *relative* changes between different cohorts of animals, or during longitudinal studies of individual specimens. When the slope is 1 and y-intercept is zero, that data may be considered fully quantitative with a reliable output of fat mass as compared to QMR. This concept will be applicable throughout the discussion of *in vivo* data sets that plot microCT and QMR data. In the case of this *in vitro* study, the linearity of both the 0.2 and 0.4 mA microCT current parameters would ultimately enable longitudinal studies in mice. However, the 0.2 mA setting was subsequently used *in vivo* because of its y-intercept value closer to 0, and also because it imparts a lower radiation dose to the animal (deep dose equivalent of 150 mSv *vs.* 300 mSv with higher current). In general, the values at each point were up to 1 g above or below the mark of the actual oil mass, for both QMR and microCT. Thus, the practical limit of precision for these techniques may be considered 1 g, and physical changes below that threshold will be difficult to measure without larger sample sizes. In summary, these *in vitro* results validate the use of both microCT and QMR for quantitative fat measurement across the range of values that correspond to adipose in living mice.

### 4.2. In Vivo Adipose Studies

The calibration of the microCT method based on the QMR method included several parameters such as the image resolution, the segmentation ranges for adipose tissue and the effect of median filter usage. Additional analysis was conducted on segmenting lean tissue using the microCT method and how effective the microCT and QMR methods are as means to estimate total body mass. Using all of the data obtained through the calibration of the imaging parameters for the microCT method, a 3D visualization of region-specific adiposity was created as a means to demonstrate the visualization of adipose tissue within the body of the mice.

The image resolution of the microCT scans led to an important distinction in its correlation with the QMR technique (Figure 2, Panel A). The strong linearity present within the lowest resolution, comprised of isotropic 500 μm voxels, will enable longitudinal tracking of relative changes in fat composition over time in living animals in a semi-quantitative fashion. Thus, even users with older, lower resolution CT systems may utilize fat segmentation techniques during longitudinal studies of mouse adipose. However, the 0.93 slope indicated that the data will not yield a perfect track with values obtained from QMR. Data reconstructed with a voxel size of 125–250 μm had a slope of 1 and y-intercept less than 1, indicating perfect linearity within the accuracy limit of 1 g noted during the *in vitro* studies. Taken together, these data indicate that fat segmentation may be performed within the 125–500 μm window to provide a valuable metric with which to track adipose changes occurring in mice over several weeks.

For the segmentation range of −300 to −50 HU, the R^2^ value of 0.97 and slope of 0.99 ± 0.03 demonstrate the validity of the microCT method for body fat quantification in mice over a wide range of body compositions (Figure 2, panel B). The y-intercept value of −0.64 does indicate a slight underestimation of fat values relative to QMR, and, as noted before, limits the overall measurement resolution to changes on the order of 1 g. The segmentations were also performed in the range of −200 to −50 HU to yield a linear correlation with an R^2^ value of 0.97. While this range would yield measurements to accurately track the relative weight changes in mice over time, the resulting slope of 0.8 caused the fat measurements to be underestimated when compared to the QMR-derived values. The expanded HU range of −300 to −50 HU was able to capture more of the adipose tissue as judged by comparison with QMR, and is the optimal range when data is collected with a 45 kVp energy, 0.2 mA current and 250 μm isotropic voxel size after reconstruction.

The decision to use the median filter on the microCT scans depends on the user's main goal; if correlative results with regards to the QMR-obtained values are required, the median filter is not recommended (Figure 2, panel C). The underestimation of the microCT adipose tissue mass values seen in the median filtered (−2.4 g) *versus* the unfiltered data sets (−0.64 g) is the primary reason the median filter would not be recommended for precise measurements. However, if *relative* changes in adipose tissue mass are the focus in a longitudinal study, the use of the median filter will not have any effect in terms of the linearity of the data as R^2^ values of 0.97 and slopes of 0.99 and 1.06 illustrate the linearity of the data.

A separate study of a heterogeneous population of 17 mice (male and female *Id2*−/− or *Id2* WT mice aged 3–9 months, bred on a mixed genetic background) was conducted to challenge the optimum microCT scan and analysis parameters gleaned from studies with a homogenous female nude mouse population. As *Id2*−/− mice exhibit an adipogenic phenotype [17,18], this also allowed for a broader range of adipose mass to be evaluated within a single population. Thus, microCT data were gathered using a 45 kVp energy and 0.2 mA current, with an output voxel size of 250 μm. The data were subsequently analyzed with a segmentation range of −300 to −50 HU and no data filtration. As noted in Figure 4, the data have excellent linearity (R^2^ = 0.97), a slope of 1 and a y-intercept of 0.01, indicating a solid alignment between the microCT and QMR values for this mixed mouse population. These data confirm the robustness of the microCT method for gathering adipose values from living mice with a range of body compositions.

### 4.3. In Vivo Lean Tissue Measurements

The linear slopes of 0.95–0.97 found within the lean tissue data show that its estimation may be made as a semi-quantitative measurement to evaluate relative changes during longitudinal microCT body composition studies. Additionally, the y-axis intercept values of −0.70, −1.52 and −2.50 demonstrate some underestimation of lean values obtained using microCT scans when compared to the QMR method. In this case, the resolution of the reconstruction does appear to be an important factor in best aligning the microCT and QMR data, with the 125 μm data yielding the best results. Nevertheless, the R^2^ values of 0.84–0.86 illustrate a weaker correlation between the microCT derived lean tissue mass values and those obtained by the QMR. These results indicate that microCT, at least using the acquisition and analysis settings attempted here, will provide a functional avenue for making semi-quantitative lean tissue mass measurements.

### 4.4. Total Body Mass

Total body mass was calculated from data obtained via microCT and QMR to determine the degree of difference between each method and measurements obtained on a balance. QMR had an almost negligible difference of −3%. Meanwhile the 500, 250, and 125 μm voxel microCT data showed a considerable difference of −16%,−11%, and −6% respectively, when the skeleton was independently segmented and added to the body mass total. The lack of the mouse tails in the microCT images contributed −2% to each of these values. The more accurate masses from the QMR method can be attributed to its precision in measuring lean tissue, which was the primary source of underestimated body weight for microCT. Nonetheless, the precision of the microCT method for adipose and total body mass measurement is essentially equal to that of QMR when the highest resolution (125 μm) is used, skeletal components are added, and the mouse tail is excluded from the calculations.

### 4.5. Visualization of Adipose Tissues

A major advantage of the microCT method is that whole animal adipose biodistribution may be directly visualized in three dimensions. Further, researchers may also identify and measure the adipose within anatomical sub-regions, like the gonadal or visceral areas. By calibrating the microCT parameters to better correlate with the values obtained by the QMR method, the 3D adiposity visualization and region-specific measurement are optimized to depict the distribution of fatty tissue within the whole mouse body. By visualizing the exact locations of body fat within the specimen, researchers will be better suited to study how this adipose tissue affects other physiological functions in nearby organs. Furthermore, the region-specific adiposity measurements hold great potential as an alternative means to analyze both distribution and change of fat at specific sites, during longitudinal studies. The increased number of obesity studies expected in the near future will undoubtedly result in innovative uses for this ability to visualize adipose tissue in 3D, which is not offered by other methods of body composition analysis.

## 5. Conclusions

MicroCT methods for adipose segmentation and measurement in mice were compared in head to head studies with quantitative magnetic resonance. Taken together, our results indicate that microCT analysis of fatty tissue is optimized when data is acquired with settings of 45 kVp, 0.2 mA, 400 projections, and an output resolution equal to or less than 250 μm. Subsequent analysis with a segmentation range of −300 to −50 HU and no data filtration yielded results with the optimum correlation to QMR. While the linearity of the microCT measurements of lean tissue mass was not as high as for adipose, the method is still reliable in detecting changes in its mass. It is important to note that previous studies stress the value and accuracy of the QMR technique for use in tracking changes in body composition between separate groups or in a longitudinal study [8–10,22–30]. Because the validation and calibration of the microCT protocol was completed based on the QMR data, this ensures that microCT is a solid choice for similar experiments in mice. Nevertheless, the microCT method holds a significant advantage by enabling both the visualization of the biodistribution of adipose and the ability to perform region-specific measurements as needed. In addition, the low technical barrier to access the equipment, general ease of use and non-invasive nature of this method are added benefits. In order to establish better pre-clinical models of the numerous clinical disorders caused by overweight and obesity, researchers may rely on microCT body composition analysis to advance their longitudinal study.
